# In vitro culture of rat hair follicle stem cells on rabbit bladder acellular matrix

**DOI:** 10.1186/s40064-016-3152-y

**Published:** 2016-08-31

**Authors:** Jia Li, Wenguang Wang, Hengqing An, Feng Wang, Mulati Rexiati, Yujie Wang

**Affiliations:** Department of Urology, The First Affiliated Hospital of Xinjiang Medical University, Address: No. 137, Liyushan Road, Urumqi, Xinjiang Uygur Autonomous Region China

**Keywords:** Hair follicle stem cells, Bladder acellular matrix, Biocompatibility, Culture

## Abstract

**Background:**

The aim of this work was to create a xenogeneic cell scaffold complex with rabbit bladder acellular matrix and rat hair follicle stem cells, to study the feasibility of construct tissue engineer bladder through biocompatibility of hair follicle stem cells and heterogeneous bladder acellular matrix.

**Material and Methods:**

New Zealand rabbit bladder acellular matrix was prepared. Scanning electron microscope and Masson staining were used to analyse the acellular material. Two-steps precipitation method was used to place the third generation of hair follicle stem cells onto the surface of the bladder acellular matrix. The in vitro cell growth on the scaffold complex was regularly monitored through an inverted microscope. Cell growth curve was established and histological examination and scanning electron microscopic were used to analyse the progresses of the cell growth on the matrix material.

**Results:**

The prepared bladder acellular matrix was white, translucent and membranous. It possessed a fibrous network and collagen structure without any significant cell residues as displayed by the scanning electron microscope, and Masson staining. After 48 h of culture, observation by inverted microscope showed that the hair follicle stem cells grew well around the bladder acellular matrix. After 1 week of culture, scanning electron microscopy showed that the hair follicle stem cells spread and adhered on the surface of the scaffold.

**Conclusions:**

The in vitro culture of rat hair follicle stem cells and the rabbit bladder acellular matrix possessed a good biocompatibility, which provides a good experiment support for hair follicle stem cells to repair the bladder defects disease.

## Background

Congenital malformations, inflammations, tumors and trauma can lead to the loss of bladder tissues structure or function, causing great suffering to the patients. In severe cases, they can lead to the decline in renal function and kidney failure. Usually, autologous non-urologic tissue or synthetic polymeric materials were used to repair or replace the bladder defect. However, since these materials cannot fully replace the function of the original tissues and organs, they can result in several adverse effects (Sumino and Mimata 2013; Xie et al. 2015; Kulikov et al. 2015; Alberti 2013; Vahabi and Drake 2015). Moreover, the limited resource of autological non-urologic tissues represents an additional obstacle. Bladder acellular matrix is a natural extracellular biomaterial which retains only the low antigenic substances including, but not limited to collagen, proteoglycan and glycoprotein. Therefore, xenogenic bladder acellular matrix used as a scaffold for bladder tissue engineering became the focus of the medical research (Liu et al. 2010; Corona et al. 2014). In this experiment, rat hair follicle stem cells, which possess the potential to differentiate into urinary tract epithelial cells and smooth muscle cells (Najafzadeh et al. 2013), were used to create a cell/scaffold complex in vitro. Thus, our present study may provide a useful alternative for bladder repair. The experiments were conducted from August 2015 to October 2015 in the Clinical Research Institute of the First Affiliated Hospital of Xinjiang Medical University.

## Methods

### Experimental animals

Five males and females SD rats, weighing approximately 200 g, 1.5 months of age, and two New Zealand rabbits, 3–5 months of age, weighing approximately 2.5 kg, were provided by the Animal Center of Xinjiang Medical University. The experiment was approved by the Animal Ethics Committee of the First Affiliated Hospital of Xinjiang Medical University (Approval Number: IACUC-20150707002).

### Reagents

Masson stain Kit (Jiangcheng Corporation, China), trypsin and EDTA digestion solution (Solarbio Corporation, China), Dispase II (Roche Corporation, Switzerland), top grade fetal bovine serum (Sijiqing Corporation, China), K-SFM culture media (Gibco Corporation, USA), type IV collagen (Sigma Corporation, USA).

### Experimental methods

#### Rabbit bladder acellular matrix preparation

The rabbit was sacrificed by air embolism. An incision in the abdominal midline was made, the bladder was removed and the fat and the fascia tissue around the bladder were removed. The bladder was rinsed 3 times in D-Hank’s buffer containing 10 % streptomycin, and then stored at 4 °C in D-Hank’s buffer. The bladder wall was incised longitudinally with the mucous membrane side up. A blade was used to carefully remove the mucous membrane and the submucosa layer was removed under the microscope. The submucosa layer was cut into 1 × 1 cm size pieces and washed 3 times with sterile PBS. The tissues were soaked and stirred in 100 mL PBS containing 0.1 % sodium azide at 250 rpm/min at room temperature overnight. Next, the tissues were rinsed with sterile PBS and placed in 100 mL of 0.5 mmol/L EDTA + 0.4 % trypsin solution, and stirred at 250 rpm/min at 37 °C for 5–6 h for digestion. Subsequently, they were rinsed with sterile PBS and placed in 100 mL of 1 mol/L NaCl solution containing DNase-I 4000 kU and stirred at 250 rpm/min at 37 °C for 6–8 h to completely digest the cells and release the cell components. The tissues were placed in 100 mL solution containing 4 % sodium deoxycholate and 0.1 % sodium azide and stirred at 250 rpm/min at room temperature for 6–8 h to dissolve the bilayer lipid cell membrane and the intracellular lipid membranes. They were rinsed 3 times in sterile PBS containing 5 % penicillin–streptomycin and placed flat into the well of a 24-well plate. The plate was placed in a vacuum freeze dryer for 6 h. The freeze-dried bladder acellular matrix was sterilized by cobalt-60 radiation with radiation dose of 25 kGy and stored at 4 °C. Scanning electron microscope and Masson staining were used for the observation of the de-cellular effect of the bladder acellular matrix.

#### Rat hair follicle stem cell culture

Single hair follicle tissue from SD rat whisker skin was dissected using microforceps under the microscope. The separated hair follicle tissues were placed in 0.25 g/L Dispase II and digested for 2 h at 37 °C on a shaker. The tissues were rinsed 3 times with PBS and were digested in a 1:1 solution of trypsin (2.5 g/L) and EDTA (0.2 g/L) for 1 h at 37 °C on the shaker. After filtration using a 200 mesh steel sieve, the mixture was centrifuged at 1500 rpm/min for 10 min. The supernatant was removed and the cells were re-suspended in 10 % fetal bovine serum and serum-free Keratinocytes medium. The cells were placed in collagen IV coated flasks, and stored in an incubator for 30 min. The non-adherent cells were removed. The remaining cells were stored in the incubator at 37 °C with 5 % CO_2_. The culture medium was changed once every 2–3 days until the cells reached 70–80 % confluence. Trypsin/EDTA was used to passage the cells to new flasks. The third generation of hair follicle stem cells were collected after days 5, washed with PBS, and divided into several fractions of 5 × 105 cells/100 μL. Each sample was measured in triplicate. PE-labeled anti-CD34, and FITC-labeled anti-CD29 were added into the suspension to a final concentration of 5 μg/mL and incubated for 30 min at 4 °C in the dark. The cells were washed with PBS twice and analyzed by flow cytometry. Fluorescence-labeled IgG isotypes were used as the control. The hair follicle stem cells were identified after morphological observation and analysis of specific surface markers. Inverted microscope was used for the cell morphology observation of the hair follicle stem cells. Flow cytometry was used to identify CD34 and β1 integrin (CD29) expression.

#### In vitro co-culture of the hair follicle stem cells and the bladder acellular scaffold

The prepared rabbit bladder acellular matrix was rinsed with sterile D-Hank’s buffer 3 times and K-SFM medium was added to pre-wet the matrix for 24 h. The K-SFM medium was removed and the prepared bladder was kept in plane condition. K-SFM medium containing 10 % of fetal bovine serum was added to the prepared bladder to pre-wet it for 2 h. Third generation of hair follicle stem cells were cultured in complete K-SFM medium containing 10 % of fetal bovine serum at 37 °C with 5 % CO_2_ until the cells were 80 % confluent. Cells were digested using 0.125 % trypsin/0.01 % EDTA. Cells were counted and 1 × 10^9^/L cells were suspended in complete K-SFM medium containing 10 % fetal bovine serum. Subsequently, 0.5 mL cell suspension was slowly added to each well, pointing the pipette tip to the center of the 24-well plate containing the pre-wetted scaffold. Same amount of cell suspension was added to an empty well as control. The plate was incubated at 37 °C with 5 % CO_2_ for 30 min, and then 0.5 mL cell suspension was additionally added to each well and incubated for additional 30 min. Next, 1 mL complete K-SFM containing 10 % fetal bovine serum was added and the plate was incubated at 37 °C with 5 % CO_2_. From the second day onward of the in vitro co-culture of the cell/scaffold complex, cells from 4 randomly selected wells were counted following digestion with 0.125 % trypsin/0.01 % EDTA and centrifugation. The cell counting was made for 10 subsequent days. The cell growth curve was plotted and compared with the control growth curve. Inverted phase contrast microscopy, scanning electron microscopy and histology were used for the morphology observation of the cell/scaffold complex and to plot the cell growth curves.

## Results

### Structural features of the bladder acellular matrix

**Fig. 1:**
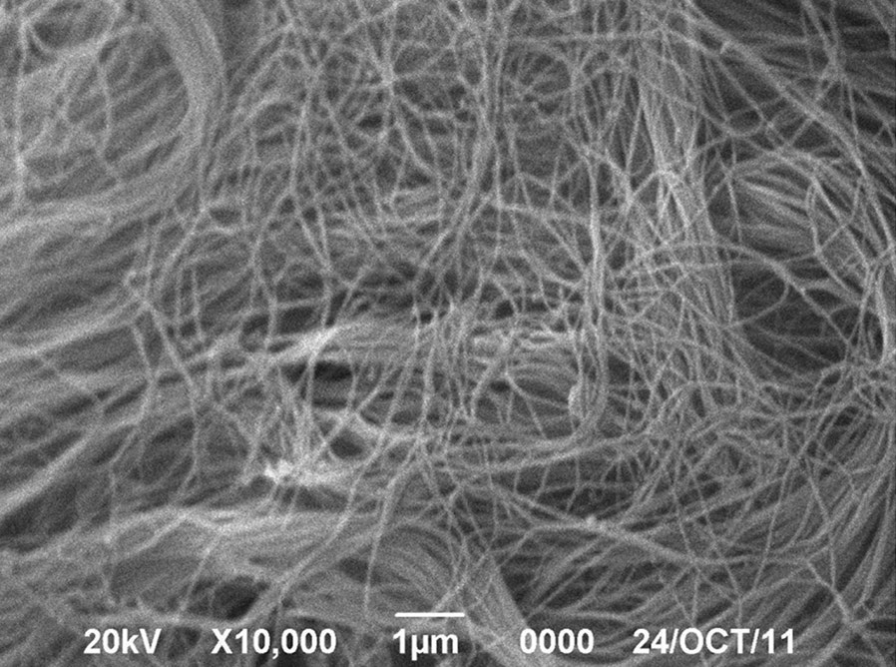
Scanning electron microscopy demonstrated the bladder acellular matrix had fiber mesh structure, but no residual cells (×10 000)

**Fig. 2:**
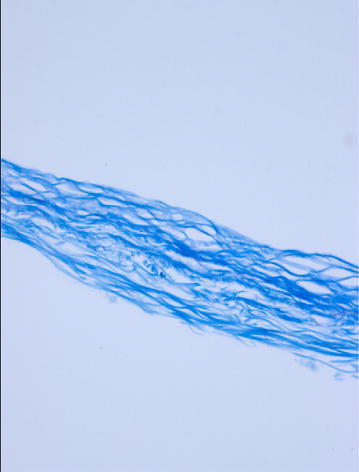
Masson staining showed the bladder acellular matrix presented with loose blue collagen fiber structure, and had no residual cells (×200)

The prepared bladder acellular matrix was white translucent and membranous, with a thickness of approximately 0.5 mm and possessed a certain elasticity and toughness. The bladder acellular matrix formed a sheet as a consequence of rehydration after lyophilisation, but wrinkles appeared when it was not immersed in the solution. Scanning electron microscope showed that the bladder acellular scaffold possessed a reticular fiber structure made of collagen with visible irregular voids, and no cell residues on the surface (Fig. 1). Masson staining of the bladder acellular matrix showed a thin layer of homogeneous blue-staining of collagen fibers with no residual cells (Fig. 2).

### Hair follicle stem cells culture and identification

**Fig. 3:**
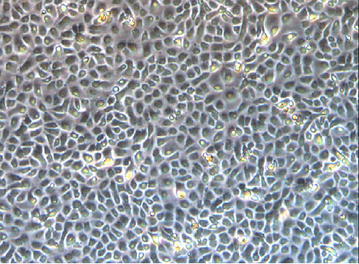
Passage 3 hair follicle stem cells cultured for 7 d showed a typical “aura” shape under the microscope (×50)

**Fig. 4:**
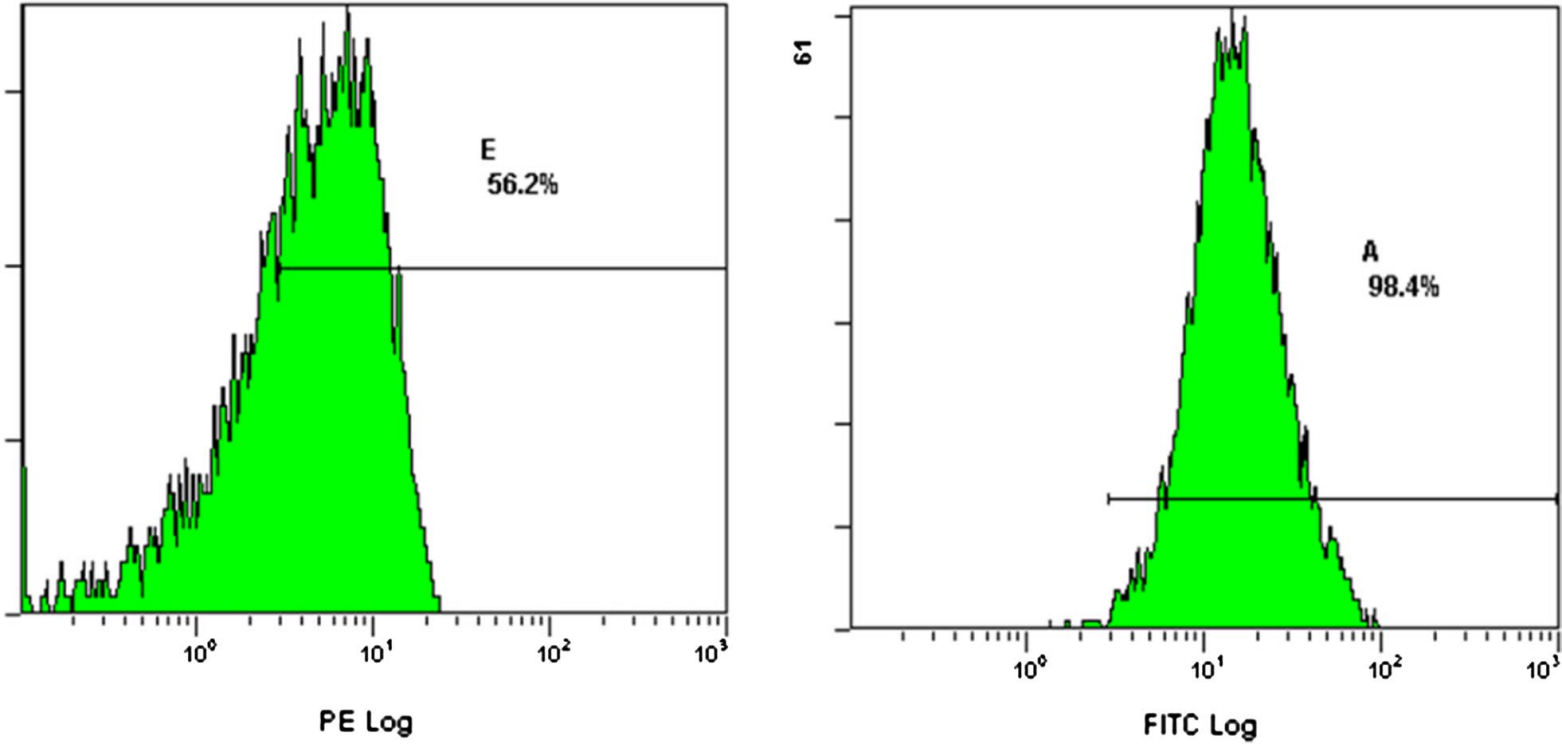
PE-labeled CD34 and FITC-labeled β1 integrin (CD29) of passage 3 hair follicle stem cells showed high expression

The hair follicle stem cells were small, polygon shaped and adherent after 48 h culture, as well as surrounded by some fibroblast cells. High purity hair follicle stem cells were obtained thanks to their ability of fast adhesion to collagen IV during cell passage compared to the fibroblasts. The third generation of hair follicle stem cells can reach 100 % confluence after 7 days of culture (Fig. 3). The expressions of CD34 and β1 integrin (CD 29) of the third generation of the hair follicle stem cells were increased after 5 days of culture (Fig. 4).

### In vitro co-culture of the hair follicle stem cells and bladder acellular matrix

**Fig. 5:**
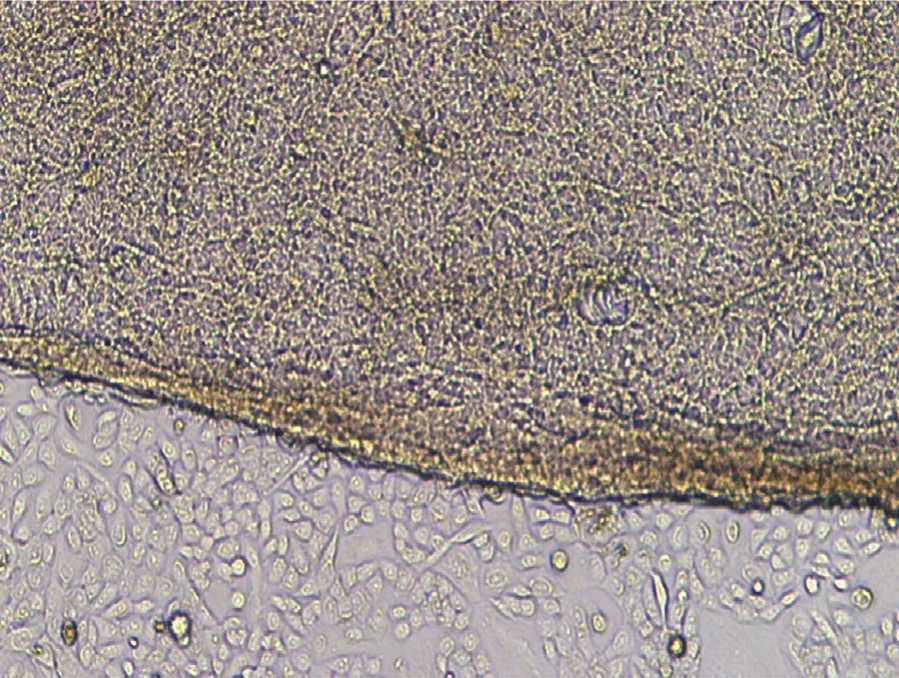
Morphology of co-cultured hair follicle stem cells with bladder acellular matrix at 48 h observed under inverted phase contrast microscope (×50)

**Fig. 6:**
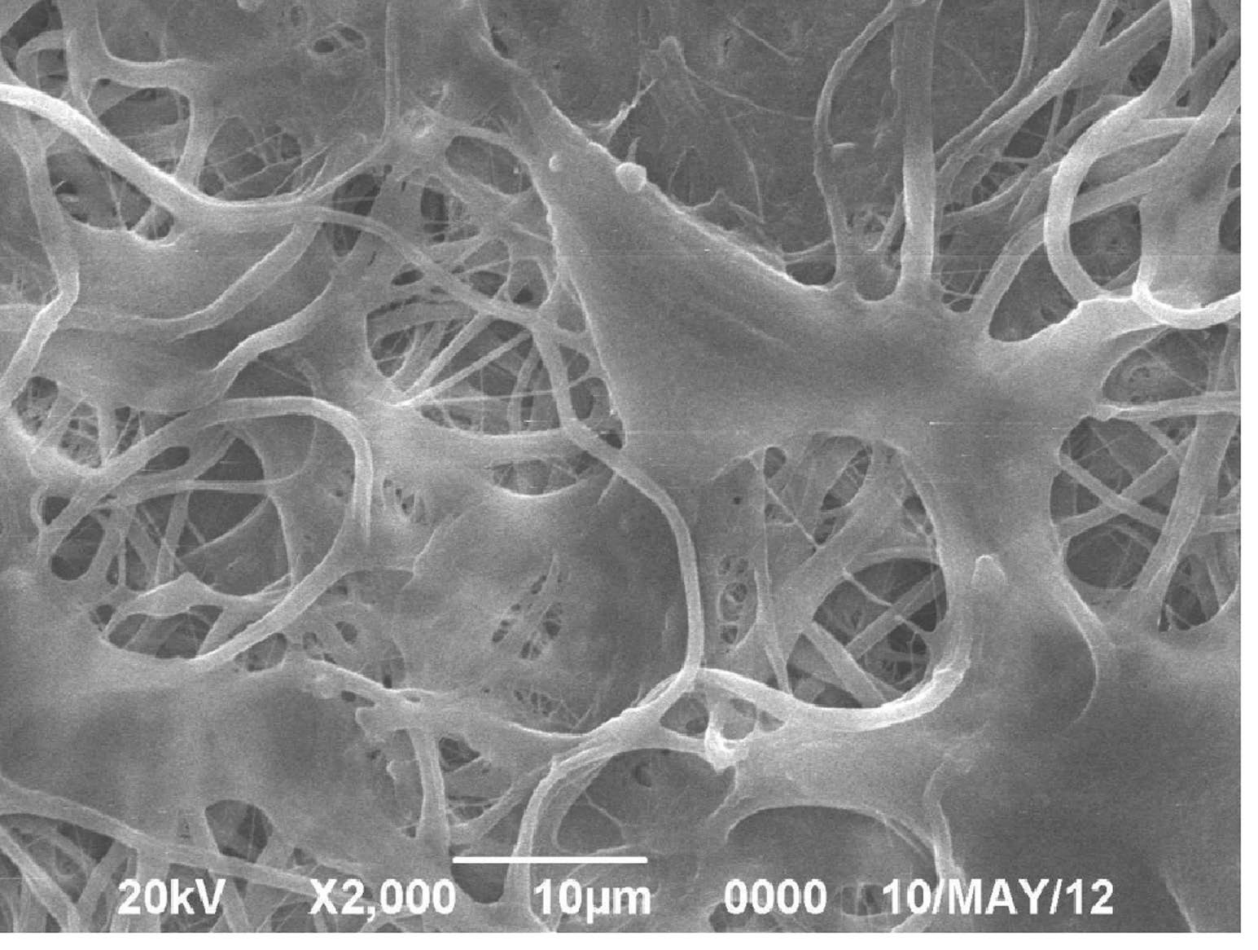
Morphology of co-cultured hair follicle stem cells with bladder acellular matrix at 48 h observed under scanning electron microscope (×1000)

Two hours after the second inoculation of the hair follicle stem cells, inverted phase contrast microscopy showed a large amount of round or oval shaped cells adhering on the scaffold and the surrounding areas at the bottom of the culture flask. Inverted phase contrast microscope showed a spindle shape and the same alignment direction of the cells adhered on the scaffold after 48 h of culture. The cells surrounding the scaffold were in good conditions and forming the typical “paving stone” (Fig. 5). After 48 h of co-culture of the cell/scaffold complex in vitro, the cells showed a plump morphology and they formed a monolayer on the surface of the scaffold as observed under the scanning electron microscope. The cells and the scaffold adhered tightly as a result of the matrix secreted by the cells, as well as the adhesion between cells (Fig. 6).

### Cell growth curve of the cell/scaffold complex

**Fig. 7:**
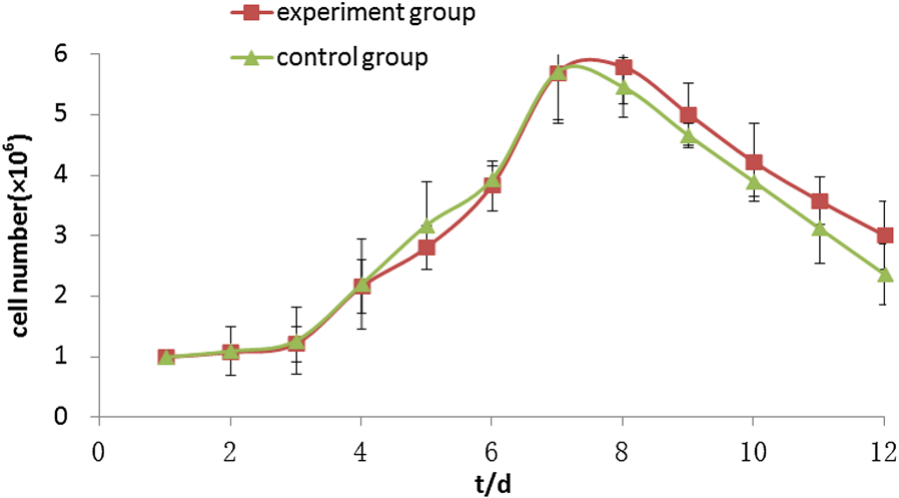
Cell growth curve of the composite material

**Fig. 8:**
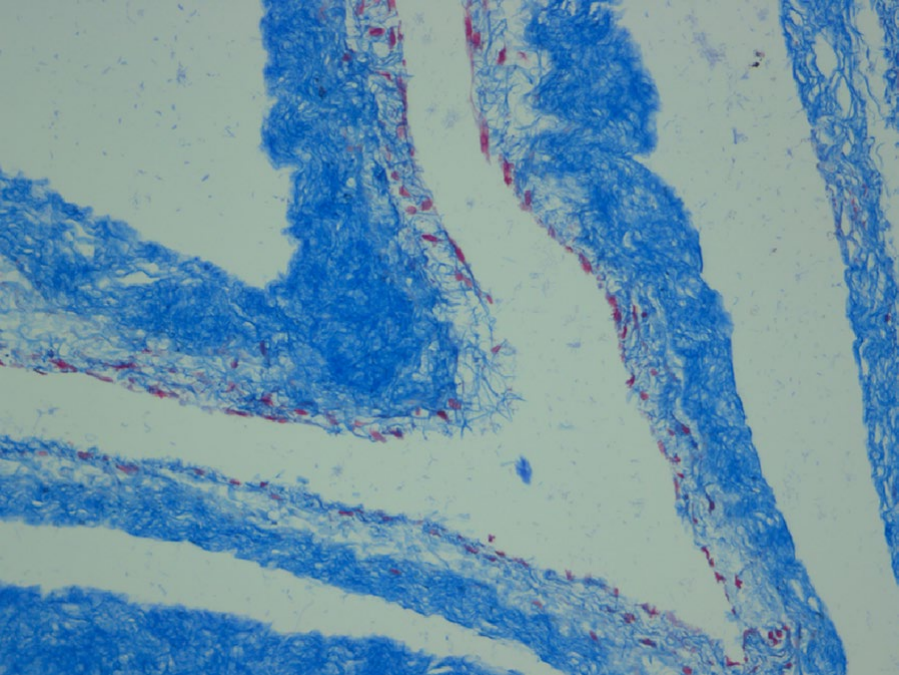
The Masson staining of hair follicle stem cells co-cultured with bladder acellular matrix for 1 week (×100)

The growth curves of the experimental group and the control group were basically the same,There was no statistical difference between the two groups (*P* *>* 0.05). Plateau phase was reached 7–8 days after the cell inoculation, indicating that the cells grew well on the scaffold surface and possessed a great biocompatibility (Table 1; Fig. 7). After 7 days of culture with fresh K-SFM complete media replacement every 48 h, the in vitro co-cultured cell/scaffold complex was fixed by paraformaldehyde and stained by Masson staining and observed under the light microscope. One to three layers of cells could be seen to adhere on the surface of the scaffold (Fig. 8).

**Table 1:** Statistical analyses for cell growth of the composite material

t/d	1	2	3	4	5	6	7	8	9	10	11	12
Experiment group (cell number/10^6)^	1.00	1.08	1.22	2.16	2.81	3.83	5.69	5.79	5.01	4.22	3.58	3.01
Control group (cell number/10^6)^	1.00	1.10	1.27	2.20	3.18	3.94	5.71	5.46	4.66	3.90	3.13	2.37
p		0.94	0.90	0.94	0.47	0.71	0.98	0.50	0.33	0.46	0.32	0.22

## Discussion

With the development of tissue engineering in recent years, the superiority of the bladder acellular matrix over other biological material has been demonstrated in a variety of experiments (Pokrywczynska et al. 2015; Zhao et al. 2015). The major advantages of the bladder acellular matrix are several: (1) The three-dimensional growth of the bladder acellular matrix is extremely similar to the original growth environment of the growth of the bladder. It represents a suitable environment for cell adhesion, growth, and connection between cells. (2) The degradation and absorption rates of the bladder acellular matrix match the regeneration of the bladder tissue. (3) The surface chemical properties are suitable for cell adsorption, growth and differentiation. (4) The removal of the antigen-containing cellular components leaves only the low antigenic materials such as collagen, proteoglycans, glycoproteins, which can prevent graft rejection after transplant and provide a scaffold for the regeneration of the bladder mucosa, muscles, blood vessels, and nerves (Chun et al. 2015; Song et al. 2014; Xiong et al. 2015). Atala et al. (2006) was the first reporting the usage of tissue engineered bladder in clinical research in 2006. This study is considered as the landmark in bladder reconstruction surgery by many researchers. The studies by Yang et al. (2010) suggested that bladder acellular matrix contains a variety of functional tissue regeneration and wound healing growth factors, including fibroblast growth factors (FGF), transforming growth factor β (TGF-β) and vascular endothelial growth factor (VEGF). These growth factors may play important roles in inducing the differentiation of stem cells into target cells. Subjected normal pig bladder to low permeability, repeated 80 °C freezing and thawing, DNase and RNase digestions and NaOH fragmentation method to prepare bladder acellular submucosa. The prepared bladder acellular submucosa showed a well-preserved structure and good biocompatibility. The results proved that the pig bladder submucosa could be used as a substitute material for tissue engineering repair (Lin et al. 2014).

Hair follicle stem cells are located at the hair follicle bulge and possess multi-directional differentiation ability. Through proliferation and differentiation, the stem cells can generate various cells to fulfill the organism’s need. The study of hair follicle stem cell was further developed since the cell marker CD34, K15 and the β1 integrin were identified. Compared with other adult stem cells, hair follicle stem cells have the following advantages: they are abundant, located on the surface thus easy to obtain, non-invasive, short growth cycle, and easy to expand. Hair follicle stem cells have been proved to be an excellent resource in regenerative medicine (Garza et al. 2011; Xu et al. 2013; Gola et al. 2012). Drewa et al. (2009) have implanted the rat hair follicle stem cells on the bladder acellular matrix and transplanted them back to rats with bladder wall defects to observe the bladder regeneration. The results showed that the bladder capacity was similar to the normal bladder capacity, and the shape of the bladder was regular. However, the safety of using hair follicle stem cells and related induction mechanisms need to be further studied.

The experiment methods used for the preparation of the bladder acellular matrix were based on the methods by Zhou et al. (2013) with slight modifications: microdissection was used combined with chemical decellularization, and subsequent improvements from trial and error were provided. Reagents such as sodium azide, sodium deoxycholate, DNase-I were used for the decellularization of the bladder submucosa. No trace of cells was found by scanning electron microscopy and histological tests. The complete decellularization effect can be achieved using this method several times (Hou et al. 2016). However, some of the chemical reagents used for decellularization are cytotoxic, such as the inorganic toxic sodium azide. When tissues are soaked in it for a long time, a small trace could remain into the tissue, compromising the subsequent cell adhesion and growth. Several experiments showed that cell growth and amplification efficiency are good in the groups with repeated PBS rinsing of the stem cells. Therefore, during the decellularization process, repeated PBS rinsing is essential for the preparation of the scaffold.

According to our own experimental conditions and long-term repeated experiments, a secondary sedimentation method was used, where cells were seeded on the scaffold 20 min after the first inoculation. The purpose of this method was to ensure that cells can be evenly distributed on and adhere to the scaffold, and to allow a penetration into the scaffold material. Tissue sectioning and staining showed that, while some cells penetrated into the scaffold, a large number of cells were still located on the surface of the scaffold. This was because cells were suspended on the surface of the scaffold material during static inoculation. Some cells diffused into the material with the diffusion of the liquid, but this passive inward movement is limited. Most cells were still stacked on the surface of the scaffold material by gravity, resulting in a large amount of cells attached to the surface and very few cells inside the scaffold. Because of the small size of the scaffold, the suspension volume used for static inoculation was only 1–2 mL. In order to get a higher seeding density, the concentration of the suspensions should be increased, but high concentration of cell suspension could easily lead to cell aggregation, which would block scaffold pores and prevent cells from penetrating the scaffold. As a result of the limited contact sites on the scaffold, many cells were loosely attached on the surface of the scaffold. These cells could easily detach when cultural medium was added and therefore, the inoculation rate decreased when the inoculation concentration increased. After careful consideration of both aspects and repeated experimentations, an inoculation concentration of 1 × 10^9^/L was used, and the results were satisfactory by histological examination.

## Conclusions

The growth curves of the experimental group and the control group were basically the same. Plateau phase was reached 7–8 days after the cell inoculation, indicating that the cells grew well on the scaffold surface and possessed a great biocompatibility. The experimental results showed that hair follicle stem cells and the xenogenic bladder acellular matrix have good biocompatibility, providing a favorable experimental basis for further differentiation. Additional exogenous inducing factors should be analysed for the promotion of the regeneration of the urinary tract epithelial cells, smooth muscle cells, vascular and neural tissues during the induced differentiation process of the hair follicles stem cells on the xenogenic bladder acellular matrix. Specific dosage and methods also need further investigation. The mechanism of the differentiation of the hair follicles stem cells induced by the bladder acellular matrix will be the focus of further research.
